# Implementation of a low-threshold, community-based consultation for young adults with early symptoms of mental disorders – a study protocol

**DOI:** 10.1192/j.eurpsy.2025.1979

**Published:** 2025-08-26

**Authors:** V. Komanek-Prinz, A. Schleussner, S. Hartmann, C. Diefenbach, A. Reif

**Affiliations:** 1 Department of Psychiatry, Psychosomatic Medicine and Psychotherapy, Goethe University Frankfurt, University Hospital, Frankfurt am Main, Frankfurt/Main, Germany

## Abstract

**Introduction:**

If untreated, mental health disorders are a leading cause of premature death due to physical illness and suicide. Typical onset is around the age of 15, and about 75% emerge by the age of 25. Thus early diagnostics and treatment to prevent chronic outcomes by early interventions is indispensable. We set up an intervention project in Frankfurt/Main, Germany, focusing on this vulnerable group aged 18 to 29.

**Objectives:**

The project aims to assess if a low-threshold, early-on psychosocial consultation model significantly reduces early symptoms of mental disorders among young adults and to evaluate whether community-based consultations reduce stigma and increase early service utilisation. We intend to measure changes in mental health literacy. Alongside consultations, we identify cooperation partners and give workshops to raise awareness and reduce stigma.

**Methods:**

A team of university psychologists and psychiatrists developed the project with community organisations and local foundations. Consultations take place in a non-stigmatizing, informal setting: the space is not within a clinic, centrally located and easily accessible. Services may be utilised anonymously and without registration of health insurance. We offer qualified diagnostics, brief solution-focused counselling, psychoeducation, early pharmacological treatment, group therapy and referral pathways to specialised care when needed. Using questionnaires, we will refine the program for potential up-scaling. Since September 5th, 2024, 12 patients aged between 17 and 34 years have already made use of the offer. Pre-intervention symptoms at baseline were initially assessed while post-intervention symptom assessments will take place 3 months later. Qualitative data will be analysed via thematic coding. Quantitative data for symptom alteration will be collected via Likert scales and analysed using paired t-tests and regressions. Qualitative feedback will be collected via surveys. We hypothesise that psychological well-being will improve post-intervention. Additionally, we expect an increased mental health literacy, alongside increased utilisation and acceptance of mental health services.

**Results:**

Yet to follow.

**Conclusions:**

Community-based mental health consultations represent a feasible early intervention strategy for young adults. Results are expected to support expanding such models to other community structures and refining protocols for scalability. We aim to optimise service delivery, assess long-term outcomes, and examine cost-effectiveness for potential implementation on a broader scale.

**Disclosure of Interest:**

None Declared

